# Effect of insertion route on risk of central line-associated bloodstream infection in critically ill patients

**DOI:** 10.1186/cc14156

**Published:** 2015-03-16

**Authors:** R Alhubail, N Hassan

**Affiliations:** 1 KFSH-D, Dammam, Saudi Arabia

## Introduction

Femoral, jugular or subclavian central venous catheterization (CVC) is routinely performed during the care of the critically ill. These invasive procedures contribute to additional morbidity, mortality, and costs derived from the interactions between traumatic, infectious and other complications. The aim of this study is to determine whether the subclavian, jugular or femoral central venous access (CVA) routes have an effect on the incidence of CLABSI in critically ill patients and to compare between these routes regarding major complications and ICU mortality.

## Methods

A retrospective observational study in a medical and surgical ICU in a tertiary care hospital on adult patients admitted from January 2010 to December 2013. The study enrolled 845 patients divided into 283 internal jugular CVC (IJC), 270 subclavian CVC (SCC) and 287 femoral CVC (FC) in which the catheters were inserted in the ICU by experienced physicians with at least 50 previously successful trials of central line insertion, using CVC bundle checklist. ICU length of stay, incidence of complications, APACHE II score adjusted severity and mortality were calculated for each group.

## Results

Patient and catheter characteristics including the duration of catheterization were similar in all groups. The rate of CLABSI in the IJC, SCC and FC groups was 5.8 versus 7.2 versus 3.45 per 1,000 catheter-days respectively with *P *= 0.35. ICU mortality was 134 (47%) cases of the IJC group, 108 (39%) cases of the SCC group and 113 (39%) cases of the FC group. There was no significant difference between the three groups of CVC in the incidence of CLABSI rate in the critically ill patients, and a slight increase in ICU mortality in the IJC group compared with the other two groups. Pneumothorax occurred in six (2.2%) cases of SCC and 11 (3.8%) cases of IJC with no significant difference between the two groups as the *P *value was 0.3.

## Conclusion

Site of insertion of CVC does not appear to affect the rate of CLABSI among critically ill patients. Pneumothorax was recorded in SCC and IJC groups with no statistical preference to either group.

